# Morphology and DNA sequences reveal a new species of *Clavellotis* (Copepoda: Lernaeopodidae) as a parasite of three species of sparids in the Yucatán Peninsula, Mexico

**DOI:** 10.1017/S0031182026101735

**Published:** 2026-04

**Authors:** Betzi F. Pérez-Ortega, Juan F. Espínola-Novelo, Raúl Castro-Romero, Martin Miguel Montes, Gerardo Pérez-Ponce de León

**Affiliations:** 1 Escuela Nacional de Estudios Superiores Unidad Mérida (ENES-Mérida), Universidad Nacional Autónoma de México, Municipio de Ucú, YU, México; 2 Departamento de Ciencias Naturales y Ciencias Ambientales, Facultad de Ciencias del Mar, Universidad de Antofagasta, Antofagasta, Chile; 3 Centro de Estudios Parasitológicos y Vectores (CEPAVE), Consejo Nacional de Investigaciones Científicas y Técnicas, Universidad Nacional de la Plata, Comisión de Investigaciones Científicas de la Provincia de Buenos Aires, La Plata, BA, Argentina

**Keywords:** integrative taxonomy, Mexico, phylogeny, Sparidae, Yucatán

## Abstract

Species of genus *Clavellotis* (Castro-Romero & Baeza-Kuroki, 1984) are parasites of marine fishes across the world. During the course of a survey on the metazoan parasites of marine fish across the Yucatán Peninsula, Mexico, specimens of a lerneapodid copepod consistent with the concept of *Clavelotis* were collected from the gills of three species of sparids, and were described as a new species using morphological and molecular characters. *Clavellotis mayae* n. sp. represents the second species of the genus reported in Mexican coastal waters. The new species morphologically resembles *C. dubius* and *C. sebastidis* in the trunk shape but can be readily distinguished by having a short maxilla which is separated to its distal end, a sub-oval and conspicuously larger aliform process, and a distal margin of the trunk bearing pronounced subcircular flaps covering the attachment sites of the egg sacs. The new species further differs from all other known congeners by having a short genital process and mandibles without secondary dentition. Molecular analyses through 28S rDNA and *cox*1 sequences further corroborate all these morphological distinctions and support the taxonomic placement of the new species within *Clavellotis*. The relationships of this species with other congeners are discussed in light of molecular evidence.

## Graphical abstract

**Figure:**
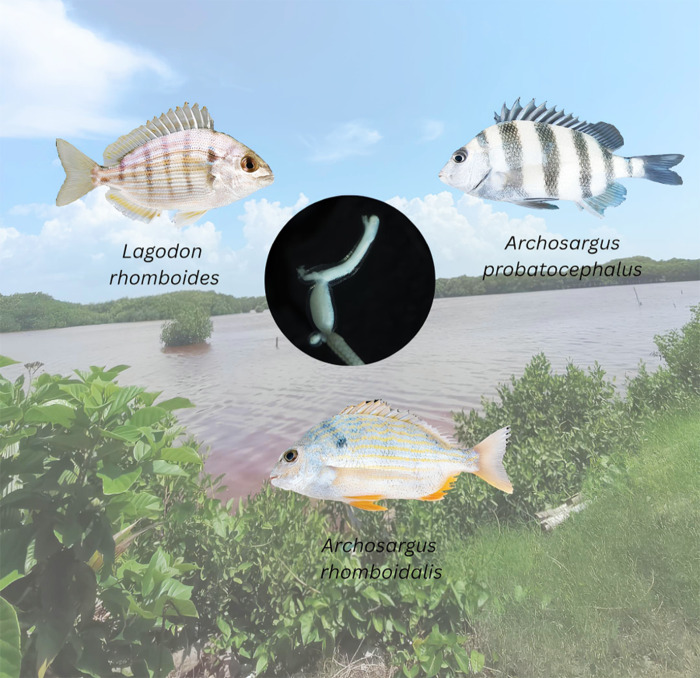

## Introduction

The genus *Clavellotis* (Castro-Romero & Baeza-Kuroki, 1984) currently contains 14 species occurring in marine fishes across the world (Castro-Romero et al., 2025). However, only 1 congeneric species has been previously reported in Mexico, *C. sebastidis* Castro and González (2005), from the gills of *Sebastes miniatus* on the Pacific coast (Rodríguez-Santiago et al., 2014, 2015, 2016; Raupach et al., 2015). Still, there are no records of *Clavellotis* on marine fishes across the Atlantic coast of Mexico.

During the course of a survey on the metazoan parasites of marine and estuarine fish of the Yucatán Peninsula, specimens of a lernaeopodid copepod were collected from the gills of three species of sparids, i.e. *Lagodon rhomboides* (L.), *Archosargus rhomboidalis* (L.) and *Archosargus probatocephalus* (Walbaum) in 2 localities. These specimens were consistent with the concept of *Clavellotis* and represented an undescribed species. The new species is described herein by using morphological and molecular characters, and its position within the phylogenetic tree of the genus is discussed.

## Materials and methods

### Sample collection

**Figure 1.**
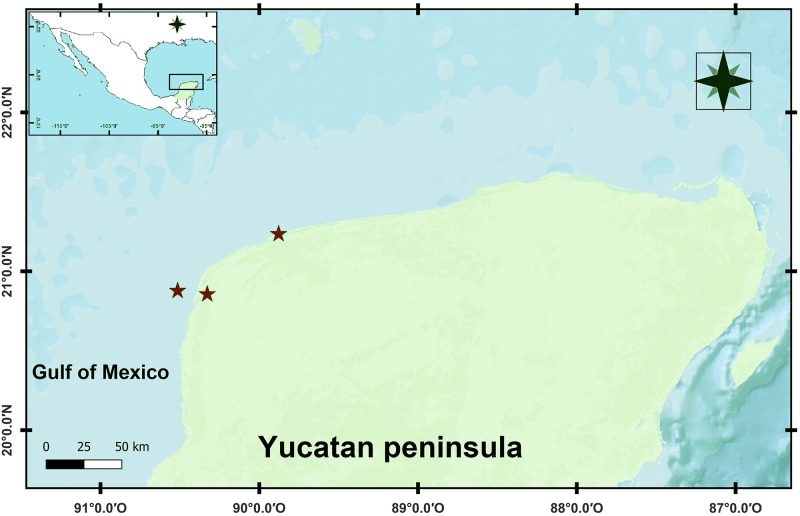
Map of the Yucatán Peninsula, Mexico showing the sampling localities, represented with a red star. Figure 1 long description.

Between 2022 and 2025, 120 individuals of 3 species of sparids, namely *L. rhomboides* (*n* = 60), *A. rhomboidalis* (*n* = 31), and *A. probatocephalus* (*n* = 29) were collected from 2 localities of the Yucatán Peninsula, La Carbonera lagoon (21°13’–21°14’ N; 89°52’–89°54’ W), and Celestún lagoon (21°13’54’’ N; 89°53’25’’ W); some specimens were also sampled from offshore Celestún (20°52’05’’ N; 90°29’08’’ W) (Figure 1). Fish were obtained from commercial capture and, in some cases, by using seine nets. In the latter case, fish were kept alive in containers with salt water and constant aeration until necropsied; these fish were euthanized by spinal severance (pithing), following the procedures accepted by the American Veterinary Medical Association (AVMA, 2020). All individuals were dissected, and gills were examined under a stereomicroscope. Specimens were preserved in 100% ethanol for further analysis. Morphological terminology follows Kabata (1979), Castro-Romero and Baeza (1984) and Boxshall and Halsey (2004). Measurements of specimens are given in micrometres. Specimens were deposited in the Colección Nacional de Crustáceos (CNCR), Instituto de Biología, Universidad Nacional Autónoma de México, Mexico City.

### DNA extraction, amplification and sequencing

Total genomic DNA was extracted from the egg sacs of individual copepods. Each egg sac was digested overnight at 56 °C in a solution containing 10 mM Tris-HCl (pH 7.6), 20 mM NaCl, 100 mM Na2 EDTA (pH 8.0), 1% sarkosyl and 0.1 mg mL^−1^ proteinase K. Total genomic DNA was extracted using DNAzol (Molecular Research Center, Cincinnati, OH) following manufacturer’s instructions. The target genes were amplified via Polymerase Chain Reaction (PCR). The partial cytochrome c oxidase subunit I (*coxI*) gene was amplified using the forward primer LCO1490 (5’-GGTCAACAAATCATAAAGATATTG-3’) and reverse primer HCO2198 (5’-TAAACTTCAGGGTGACCAAAAAATCA-3’) (Folmer et al., 1994), using the following cycling conditions: 4 min initial denature at 94°C, followed by 34 cycles of 94 °C for 30 sec, 44–48 °C for 1 min and 72 °C for 1 min; and a final extension phase at 72 °C for 7 min. The large subunit of the nuclear ribosomal RNA gene (28S) was amplified using the forward primer 391 (5’-AGCGGAGGAAAAGAAACTAA-3’) (Nadler and Hudspeth, 1998) and reverse primer 536 (5’-CAGCTATCCTGAGGGAAAC-3’) (García-Varela and Nadler, 2005). Sequencing internal primers were 502 plus 503 (Stock et al., 2001; García-Varela and Nadler, 2005). Cycling conditions: 4 min initial denature at 95 °C followed by 30 cycles of 94 °C for 30 seg, 50 °C for 45 seg and 72 °C for 2 min; and a final extension phase at 72 °C for 7 min. Contigs were assembled and base-calling differences were resolved using Geneious Pro 4.8.4 (Biomatters Ltd). Sequences were compared to the GenBank library content with the BLAST tool, and were deposited at the GenBank database (Table 1).

**Table 1.** Collection data and GenBank accession numbers of the copepods used in this study. Newly generated sequences are shown in bold Table 1 long description.

Species	Host	Site of collection	*Cox*I	28S	Reference
*Alella canthari* (Heller, 1865)	*Spondyliosoma cantharus* (L., 1758) A	Turkey	PX243275-76		Montes et al., 2025
*Alella igillimpethu* (Erasmus et al., 2022)	*Clinus superciliosus* (L., 1758) A			OP548634-36	Erasmus et al., 2022
*Alella macrotrachellus* (Brian, 1906)	*Diplodus vulgaris* (Forster, 1801) A	Turkey	PX243277-78		Montes et al., 2025
*Simplalella igillimpethu* (Erasmus et al., 2022)	*Clinus superciliosus* (L., 1758) A	South Africa	OP548138-39		Erasmus et al., 2022
*Brachiela quaternia* (Wilson C.B., 1935)		India	OP425696		Nikhila and Kappalli, 2022^a^
*Brachiella thynni* (Cuvier, 1830)		Australia		EU118307	Hayward, et al., 2007^a^
		India	PQ686636		Swaraj et al., 2024^a^
*Brianella corniger* (Wilson C.B., 1915)	*Psammobatis bergi* (Marini, 1932) E	Argentina	PX226557		Montes et al., 2025
*Clavella adunca* (Strøm, 1762)	*Gadus morhua* (L., 1758) A	North Sea	KT208865, 702, 899		Raupach et al., 2015
				MF077820	Khodami et al., 2019^a^
*Clavella perfida* (Wilson C.B. 1915)			LC179744-46		Yanagimoto et al., 2016^a^
*Clavella stellata* (Krøyer, 1838)				DQ180339	Tjensvoll et al., 2005^a^
*Clavellisa scombri* (Kurz, 1877)	*Scomber scombrus* (L., 1758) A	Turkey	PV696745-46		Castro Romero et al., 2025
*Clavellisa ilishae* (Pillai, 1962)		India	ON023682		Nikhila and Sudha, 2022^a^
*Clavellisa obcordatus* (Rangnekar, 1957)		India	ON023683		
*Clavellomimus macruri* (Hansen, 1923)	*Macrourus holotrachys* (Günther, 1878) A	Chile		MH917255	Castro-Romero et al., 2016^a^
*Clavellotis dilatata* (Kroyer, 1863)	*Cheilodactylus variegatus* (Valenciennes, 1833) A	Chile	KX815897-900	KX815915	Castro-Romero et al., 2022
				PV696730	Castro-Romero et al., 2025
*Clavellotis dubius* (Castro-Romero et al., 2025)	*Chromis crusma* (Valenciennes, 1833) A	Chile	PV696736-41	PV698390-91	
*Clavellotis fallax* (Heller, 1865)	*Dentex dentex* (L., 1758) A	Turkey	PV696731-32		
*Clavellotis girellae* (Castro-Romero et al., 2025)	*Girella laevifrons* (Tschudi, 1846) A	Chile	PV696725-29	PV698387-89	
***Clavellotis mayae* n. sp.**	***Lagodon rhomboides* (L., 1766) A**	**Mexico**	**PX830982-88**	**PX802343-44**	**This study**
	***Archosargus rhomboidalis* (L., 1758) A**		**PX802389-92**	**PX802345-46**	
	***Archosargus probatocephalus* (Walbaum, 1792) A**		**PX800993**		
*Clavellotis pagri* (Krøyer, 1863)	*Sarpa salpa* (L., 1758) A	Turkey	PV696742-43		Castro-Romero et al., 2025
*Clavellotis sargi* (Kurz, 1877)	*Diplodus vulgaris* (Geoffroy Saint-Hilaire, 1817) A		PV696744		
*Clavellotis sebastidis* (Castro & González, 2005)	*Sebastes oculatus* (Valenciennes, 1833) A	Argentina	PV696735		
*Clavellotis strumosa* (Brian, 1906)	*Pagrus pagrus* (L., 1758) A	Turkey	PV696733-34		
*Eubrachiela antartica* (Quidor, 1906)		South Georgia	PP595770		Zheng et al. 2024^a^
Lernaeopodidae gen et sp.				KR048860	Baek and Hwang 2015^a^
*Lernaeopoda bivia* (Leigh-Sharpe, 1930)	*Galeorhinus galeus* (L., 1758) E	Uruguay	OP712629-30		Montes et al. 2022
	*Schroederichthys bivius* (Müller & Henle, 1838) E	Argentina	MN822000-01		
*Maxiclavella simplex* (Castro-Romero, 1985)	*Isacia conceptionis* (Cuvier, 1830) A	Chile	KX815901-03	KX815911	Castro-Romero et al., 2022
*Naobranchia vermiformis* (Rangnekar, 1956)		India	ON023684		Nikhila and Sudha, 2022^a^
*Neobrachiella merluccii* (Bassett-Smith, 1896)				DQ180347	Tjensvoll et al., 2005^a^
*Parabrachiella albida* (Rangnekar, 1956)				MT994259	Kappalli and Nikhila 2020^a^
*Parabrachiella anisotremi* (Castro-Romero & Baeza-Kuroki, 1989)	*Anisotremus scapularis* (Tschudi, 1846)	Chile	KX815887-90	KX815913	Montes et al., 2017
*Parabrachiella auriculata* (Castro-Romero & Baeza-Kuroki, 1987)	*Cilus gilberti* (Abbott, 1899) A	Chile	KX815906-08	KX815914	
*Parabrachiella chevreuxii* (Beneden, 1891)	*Pogonias courbina* (Lacepede, ‘ 1803) A	Argentina, Tapera de Lopez	MZ356556		Montes et al., 2022
*Parabrachiella dispar* (Castro-Romero & Baeza-Kuroki, 1987)	*Cilus gilberti* (Abbot, 1899) A	Chile	MZ356555		
*Parabrachiella exilis* (Shiino, 1956)	*Mugil cephalus* (L., 1758) A	Chile	KY026072-74		Montes et al., 2017
*Parabrachiella fasciata* (Castro-Romero & Baeza-Kuroki, 1987)	*Sciaena fasciata* (Tschudi, 1846) A	Chile	MZ356552		Montes et al., 2022
*Parabrachiella hugu* (Yamaguti, 1939)		South Korea	KT030285	KR048861	Baek and Hwang, 2015^a^
*Parabrachiella kabatai* (Luque & Farfan, 1991)	*Isacia conceptionis* (Cuvier, 1830) A	Chile	KY026075-78		Montes et al., 2017
*Parabrachiella merluccii* (Bassett-Smith, 1896)	*Merluccius merluccius* (L., 1758) A	North Sea	KT208689		Raupach et al., 2015
*Parabrachiella mugilis* (Kabata, Raibaut & Ben Hassine, 1971)	*Chelon auratus* (Risso, 1810) A	Turkey	MZ356558		Montes et al., 2022
*Parabrachiella oralis* (Castro-Romero & Baeza-Kuroki, 1987)	*Sciaena deliciosa* (Tschudi, 1846) A	Chile	MZ356553-54		
*Parabrachiella platensis* (Montes et al., 2017)	*Mugil liza* (Valenciennes, 1836) A	Argentina	KY026079-85		Montes et al., 2017
*Praeclavella applicata* (Castro-Romero & Baeza -Kuroi, 1985)	*Anisotremus scapularis* (Tschudi, 1846) A	Chile	KX815891-93	KX815912	Castro-Romero et al., 2022
*Praeclavella caudata* (Castro-Romero & Baeza -Kuroi, 1985)			KX815894-96	KX815910	
*Praeclavella nasalis* (Castro-Romero et al., 2022)	*Isacia conceptionis* (Cuvier, 1830) A	Chile	KX815904-06	KX815912	
*Pseudocharopinus bicaudatus* (Krøyer, 1837)	*Squalus acutipinnis* (Regan, 1908) E	South Africa	MZ848416-17		Dippenaar et al., 2021
*Pseudocharopinus tenshken* (Castro-Romero & Bovcon, 2025)	*Squalus acanthias* L.	Argentina	PX226555-56		Montes et al., 2025
*Pseudocharopinus malleus* (Rudolphi in von Nordmann, 1832)	*Torpedo fuscomaculata* (Peters, 1855) E *T. sinuspersici* (Olfers, 1831) E		MZ848418-19		Dippenaar et al., 2021
*Pseudocharopinus pillai* (Kabata, 1979)	*Dasyatis pastinaca* (L.)		MZ695835-36		
*Pseudocharopinus pteromylaei* (Raibaut & Essafi, 1979)	*Aetomylaeus bovinus* (Geofroy SaintHilaire, 1817) E		MZ695833-34		
*Salmincola californiensis* (Dana, 1852)	*Oncorhynchus mykiss* (Walbaum, 1792) A	North America		KY113082-83	Ruiz et al., 2017
		Russia	OQ844022		Shedko et al., 2023
*Salmincola edwardsii* (Olsson, 1869)		Norway		DQ180346	Tjensvoll et al., 2005^a^
	*Salvelinus fontinalis* (Mitchill, 1814) A	North America		KY113080-81	Ruiz et al., 2017
*Salmincola carpionis* (Krøyer, 1837)	*Salvelinus alpinus* (L., 1758) A)	Greenland	OQ843996		Shedko et al., 2023
*Salmincola markewitschi* (Shedko & Shedko, 2002)	*Salvelinus leucomaenis* (Pallas, 1814) A	Japan		LC713076-77	Hasegawa et al., 2022
		Russia	OQ843970		Shedko et al., 2023
*Salmincola siscowet* (Smith, 1874)		North America	OP830211		Marshall et al., 2023^a^
*Thysanothe eleutheronema* (Rangnekar, 1962)			MN064569		Nikhila and Sudha, 2022
		India	PQ149024		Swaraj et al., 2024^a^
Outgroup					
*Ergasilus* sp.	*Mugil liza* (Valenciennes, 1836) A	Argentina	KU557411		Castro-Romero et al., 2016
				KR048842	Baek and Hwang, 2015^a^

a Unpublished sequences available in the GenBank dataset.

### Sequence comparison and phylogenetic analysis

Sequences were aligned using MAFFT v.7 (Katoh et al., 2019) via the online server. The best partitioning scheme and substitution model were selected under the Bayesian Information Criterion (BIC; Schwarz, 1978) using the ‘greedy’ algorithm implemented in PartitionFinder v.1.1.1 (Lanfear et al., 2014). The optimal nucleotide substitution models were SYM + I + G for the first, GTR + I + G for the second, GTR + G for the third codon positions of COI, and K80 + G for 28S. Phylogenetic reconstruction was performed with Bayesian Inference (BI) in MrBayes v.3.2.3 (Ronquist et al., 2012). Two independent Metropolis-Coupled Markov Chain Monte Carlo (MCMC) analyses were run for 20 million generations, sampling every 1000 generations. Convergence was assessed by ensuring that the average standard deviation of split frequencies remained below 0.01, as recommended by Ronquist et al. (2012). Posterior Probabilities (PP) were used as measures of clade support. A majority-rule consensus tree with branch lengths was constructed after discarding the first 25% of sampled trees as burn-in. To estimate divergence among lineages, pairwise genetic distances (*p*-distance; Nei and Kumar, 2000) were calculated. Distance matrices were generated in MEGA X (Kumar et al., 2018), using 1000 bootstrap replicates and assuming uniform nucleotide substitution rates (transition + transversions).

## Results

### Systematics

Class Copepoda (Milne Edwards, 1840)

Order Siphonostomatoida (Burmeister, 1835)

Family Lernaeopodidae (Milne Edwards, 1840)

Genus *Clavellotis* Castro-Romero & Baeza-Kuroki, 1984

*Clavellotis mayae* n. sp.

### Description

**Figure 2.**
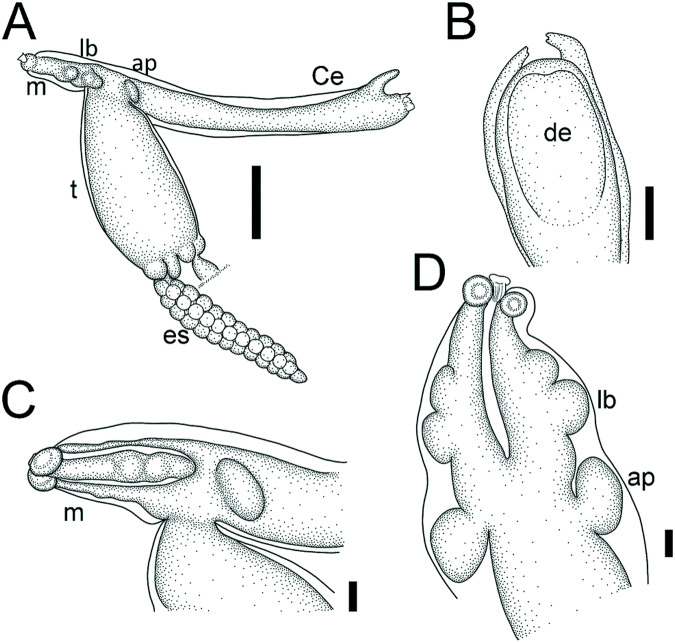
Drawings of the female of *Clavellotis mayae* n. sp. from the Yucatán Peninsula, Mexico. (A) Female, lateral view. (B) Dorsal shield. (C) Maxilla and aliform process, lateral view. (D) Maxilla and aliform process, dorsal view. Abbreviations: ap, aliform process; Ce, cephalothorax; es, egg sac; m, maxilla; lb, lateral lobes; t, trunk. Scale bars: A = 500 µm; B, C, and D = 100 µm. Figure 2 long description.

**Figure 3.**
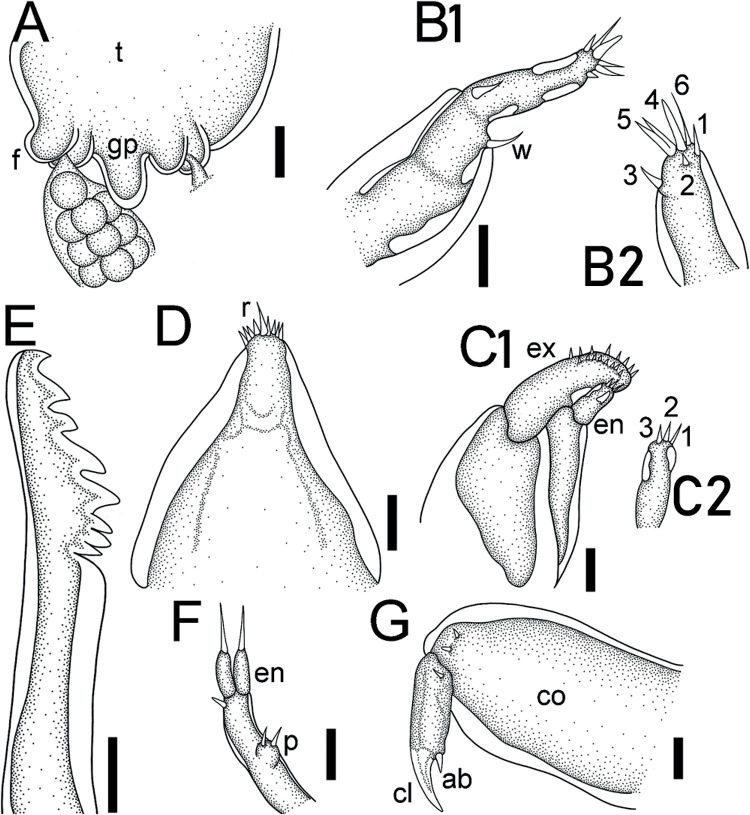
Drawings of the female of *Clavellotis mayae* n. sp. from the Yucatán Peninsula, Mexico. (A) Trunk distal margin. (B1) Antennule entire; (B2) detail of distal armature. (C1) Antenna; (C2) endopod detail. (D) Labrum ventral view. (E) Mandible. (F) Maxillule. (G) Maxilliped. Abbreviations: ab, accessory barb; cl, claw; co, corpus; en, endopod; ex, exopod; f, flaps; gp, genital process; p, palp; r, rostral seta; 1, 6, 4, 5, 3, 2, antennule armature; 3, 2, 1, endopod setate; t, trunk; w, whip. Scale bars: A = 100 µm; B, C, D and E = 20 µm; F = 10 µm and G = 50 µm. Figure 3 long description.

*Female*: Measurements (based on the holotype and 23 paratypes specimens) in micrometres (Figures 2, 3, 5A, 5B, and 5C). Cephalothorax 2217–2914 (2436; *n* = 23) length by 206–548 (398; *n* = 23) width (Figure 2A). Dorsal shield well defined, tapering posteriorly (Figure 2B). Maxilla 563–1016 (729; *n* = 22) length by 178–473 (330; *n* = 22) width; shorter than cephalothorax, reaching ca. 28.9% of its length, separated, and fused only distally; each maxilla has 2 lobes; aliform processes oval, oblique, near base of maxilla and distant from excretory duct (Figures 2A, 2C, and 2D). Trunk 1265–1912 (1,663; *n* = 24) length by 636–984 (845; *n* = 24) width (Figure 2A); distal part wider than medial and basal regions; elongated, pyriform, with a conspicuous subcircular projection (flap) covering egg-sac insertion (Figure 3A). Genital process short 155–311 (253; *n* = 22) length by 116–195 (164; *n* = 22) width, blunt distally (Figure 3A). Egg sac 782–2,760 (1980; *n* = 17) length by 183–651 (392; *n* = 17) width (Figure 2A). Antennule short 127; (*n* = 1) length, 4-segmented; basal part widened (2 segments well defined), bearing a short whip (Figure 3B1). Distal 2 segments lacking solus; terminal segment with armature: setae 1, 2, 3 (spiniform), 4 (digitiform), 5 (bifid), 6 (longest) (Figure 3B2). Antenna 219 (*n* = 1) length; sympod strong, wide; exopod longer and wider than endopod, dorsal margin with 2 rows of denticles (Figure 3C1); endopod 2-segmented, narrower, distally with 3 equal spines (1–3) (Figure 3C2). Labrum subtriangular; rostrum with a central long seta, 4 short setae on each side (Figure 3D). Mandible blade with formula P1, P1, P1, B5; lacking secondary teeth; primary dentition subequal, first 2 basal teeth longer (Figure 3E). Maxillule 101 (*n* = 1) length, lobate; endite with 2 papillae each bearing long setae and 1 shorter dorsally; palp lateral, with 2 unequal short setae (Figure 3F). Maxilliped corpus wide 190 (*n* = 1); myxal area with 1 spine; distal margin with 2 small processes (1 subtriangular, 1 blunt); subchela strong (37.5% of corpus length) with proximal seta; claw slightly curved, with short accessory barb (one-third of claw length) (Figure 3G).

**Figure 4.**
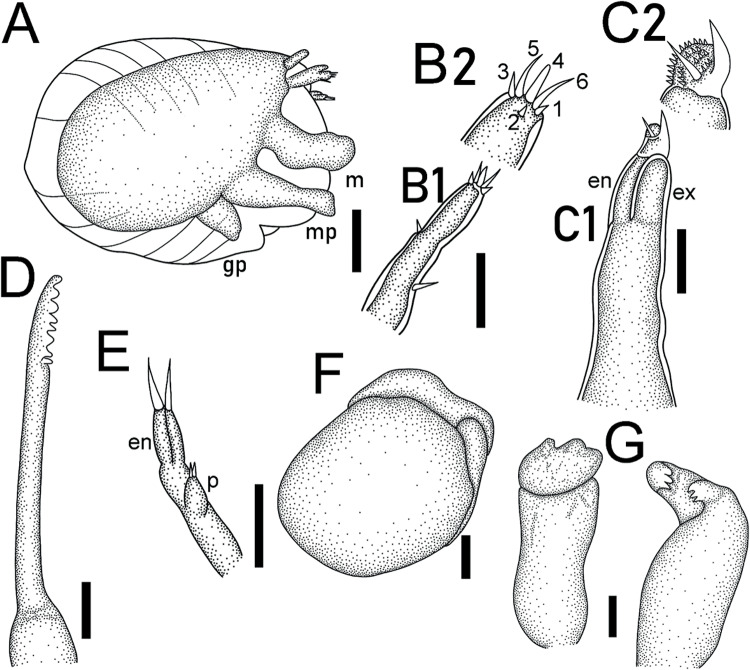
Drawings of the male of *Clavellotis mayae* n. **s**p. from the Yucatán Peninsula, Mexico. (A) Male, entire, lateral view. (B1) Antennule entire; (B2) detail of distal armature. (C1) Antenna entire; (C2) detail distal of endopod. (D) Mandible. (E) Maxillule. (F) Maxilla. (G) Maxilliped dorsal and ventral view. Abbreviations: m, maxilla; mp,  maxilliped; gp, genital process; en, endopod; ex, exopod; p, palp; 1, 6, 4, 5, 3, 2, antennule armature. Scale bars: A = 100 µm; B, C = 20 µm; D = 10 µm; E, F and G = 25 µm. Figure 4 long description.

**Figure 5.**
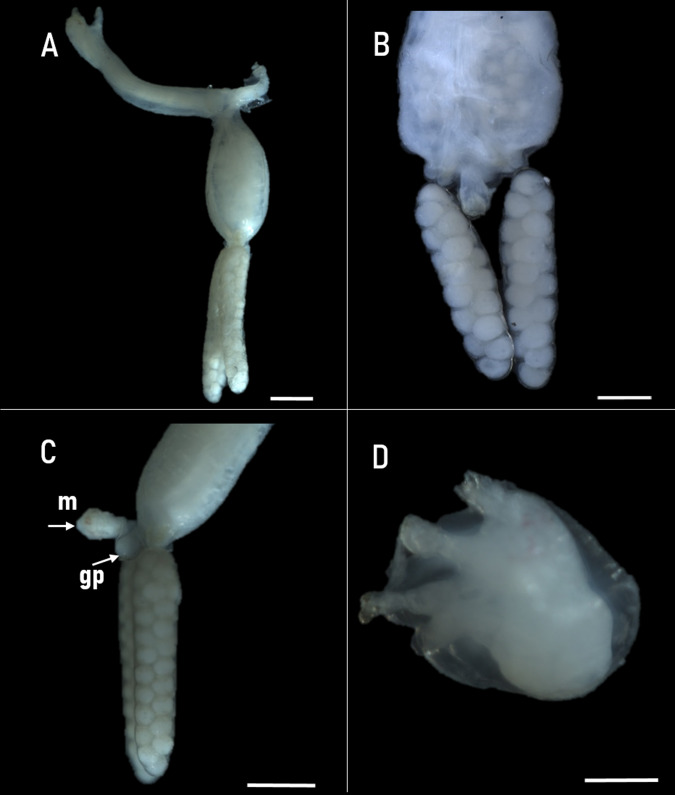
Photographs of *Clavellotis mayae* n. sp. from the Yucatán Peninsula, Mexico. (A) Female, entire lateral view. (B) Posterior end of female with egg sacs. (C) Male attached to the female genital process. (D) Male. Scale bars: A, B and C = 500 µm; D = 200 µm. Figure 5 long description.

*Male*: (based on Allotype and 6 paratypes specimens) in micrometres (Figures 4, 5C, and 5D). Usually attached near female genital process (Figure 5C). Suborbicular, longer than wide 609–792 (549; *n* = 7) length by 508–558 (486; *n* = 7) width, trunk strongly curved; genital process short, disto-ventral, near maxilliped base (Figure 4A). Antennule 41 (*n* = 1) length, uniramous (Figure 4B1), 3-segmented; basal segment with distal whip; second with short solus; distal segment with armature: setae 1, 2, 3 (spiniform); 4 (digitiform); 5 (simple); 6 (longest) (Figure 4B2). Antenna 89 (*n* = 1) length; 2-segmented base (Figures 4B1 and B2); endopod and exopod are in line with the sympod, and endopod is longer than exopod; with strong dorsal curved spine, ventral adhesion pad densely spinulose and 1 seta; exopod unarmed (Figures 4C1 and C2). Mandible very thin blade, armed distally with 8 equal teeth, lacking secondary dentition (Figure 4D). Maxillule endite with 2 papillae each bearing 1 seta; palp lateral, with 2 short distal setae (Figure 4E). Maxilla first segment globose; distal part forming curved claw (Figure 4F). Maxilliped first segment rectangular, disto-ventrally with 3 teeth; second segment short, distally armed ventrally with 3 teeth (Figure 4G); both segments aiding attachment to female genital area.

### Taxonomic summary

**Type host:** *Lagodon rhomboides* (Linnaeus, 1766)

**Other hosts:** *Archosargus rhomboidalis* (Linnaeus, 1758)

*Archosargus probatocephalus* (Walbaum, 1792).

**Type locality:** La Carbonera lagoon, Yucatán Peninsula, Mexico, Gulf of Mexico

**Other locality:** Celestún, Yucatán

**Site of infection:** gills

**Material examined**: 32 specimens from *L. rhomboides* (22 female and 10 male, 10 fish infected of 60 examined, intensity: 1–7); 45 from *A. rhomboidalis* (41 female and 4 male, 13 fish infected of 31, intensity 1–7); and 8 from *A. probatocephalus* (7 female and 1 male, 4 fish infected of 29, intensity 1–9).

**Etymology:** The specific epithet, *mayae* refers to the Maya civilization that inhabited the Yucatán Peninsula, where the host species of the copepod are widely distributed.

**Specimens deposited:** Holotype female CNCR 38370, Allotype male CNCR 38369, paratype female and male of *L. rhomboides* CNCR 38371 from La Carbonera lagoon, paratype female of *A. rhomboidalis* CNCR 38372 and paratype female of *A. probatocephalus* CNCR 38373 from Celestún lagoon were deposited in the Mexican National Crustacean Collection (CNCR).

**GenBank accession:** PX802343*–*PX802346 for 28S; PX830982*–*PX830993 for *cox*1

**ZooBank LSID:** zoobank.org:act:08155F38-6947-4A6D-84D9-D0B980CEB8F2XX

### Remarks

According to the diagnosis given by Castro-Romero and Baeza (1984), *Clavellotis mayae* n. sp. belongs to the genus *Clavellotis* by possessing a lateral process at the base of cephalothorax and by having a trunk without posterior processes, but with a genital process.

Among the currently known species of *Clavellotis*, the new species can be readily distinguished from those exhibiting a subcircular trunk, i.e. *C. dilatata* (Krøyer, 1863), *C. girellae* Castro-Romero et al. (2025), *C. tarakihi* (Hewitt & Blackwell, 1987) and *C. branchiostegui* (Yamaguti, 1939) because they lack an elongated, and pyriform to sub-quadrangular trunk. In this respect, *C. mayae* n. sp. is more similar to *C. briani* Benmansour, Ben Hassine, Diebakate & Raibaut, 2001, *C. dubius* (Castro-Romero et al. 2025), *C. fallax* (Heller, 1865), *C. pagri* (Krøyer, 1863), *C. sargi* (Kurz, 1877), *C. sebastidis* (Castro and González 2005) and *C. strumosa* (Brian, 1906). However, most of these species can be readily distinguished. For instance, the trunk in *C. fallax, C. strumosa, C. sargi* and *C. pagri* is slightly longer than wide; the trunk in *C. briani* is subquadrangular, whereas in the new species the trunk is elongated, pyriform, and possess a conspicuous subcircular projection (flap) covering the egg-sac insertion. Thus, the new species is morphologically more similar to *C. dubius* and *C. sebastidis* by the shape of the trunk.

### Comparison with C. dubius

In *C. dubius*, the maxilla is short, about half the trunk length, and lacks lateral lobes whereas in the new species, the maxilla bears 2 distinct lateral lobes. The aliform process is oval and simple in both species. The trunk of *C. dubius* lacks distal flaps over the egg-sac attachment, instead showing 2 lobes, while the new species bears subcircular flaps covering the egg-sac bases. The genital process is subrectangular and more developed in *C. dubius, vs.* short and blunt in the new species. The antennule is 4-segmented in both, but the armature differs (1, 3, 4, 5, 6 in *C. dubius*; 1, 2, 3, 4, 5, 6 in *C. mayae* n. sp.). In the antenna, *C. dubius* shows an exopod longer than the endopod, with few dorsal spines; the endopod bears 3 unequal setae. In contrast, the new species has 2 rows of dorsal spinules on the exopod and 3 subequal endopodal setae. The mandibular formula also differs: *C. dubius* shows P1S1, P1S1, P1S1, B5, while *C. mayae* n. sp. lacks secondary dentition, with the first 2 basal teeth larger than the others. The maxillule is similar in endite armature but differs in palp setation. The maxilliped of the new species bears 2-min distal processes absent in *C. dubius*. Males of *C. mayae* n. sp. are more distinctly oval (*vs*. subcircular in *C. dubius*), with maxilla and maxilliped positioned close together, and differs in claw shape and maxilliped armature. The species *C. dubius* was described from the pomacentrid *Chromis crusma* (Valenciennes, 1833) off the coast of Chile, in the Southeast Pacific, whereas the new species is described from sparids in the Gulf of Mexico.

### Comparison with *C. sebastidis*

*Clavellotis sebastidis* possess an elongated trunk, approximately equal in length to the cephalothorax, lacking lobes at the egg-sac base; whereas in *C. mayae* n. sp., the cephalothorax is longer than the trunk and bears distinct flaps over the egg-sac base. The maxilla in *C. sebastidis* is as long as the trunk and lacks protuberances, whereas in the new species it is short (about one-third trunk length), and bears paired lateral lobes. The aliform process is circular in *C. sebastidis*, oval in the new species. The antennule is 3-segmented in *C. sebastidis* but 4-segmented in the new species, and the armature differs accordingly. The antenna is similar in both, but the mandible differs: *C. sebastidis* shows P1S1, P1S1, P1S1, B4, whereas *C. mayae* n. sp. lacks secondary dentition and has the first 2 basal teeth longer. The maxillule also differs in palp setation. The maxilliped is similar in general structure, but *C. sebastidis* lacks distal processes on the corpus whereas the new species possesses 2-min distal processes on the corpus absent; in addition, *C. sebastidis* has a spinulose ventral subchela margin, which is absent in the new species. Males of *C. sebastidis* are subcircular and possess a dorsal projection, while *C. mayae* n. sp. is oval-shaped and lacks dorsal projection. The genital process is shorter and more separated in *C. sebastidis*; in the new species, it is more developed and positioned near the maxilliped. Antennule armature, mandibular dentition and maxillule setation also different. *Clavellotis sebastidis* was originally described by Castro and González (2005) from the rockfish *Sebastes oculatus* (Valenciennes 1833) off the southern coast of Argentina, and was later reported from *S. miniatus* (Jordan & Gilbert 1880) along the Northwest Pacific coast of Mexico (Rodríguez-Santiago et al., 2014, 2015, 2016).

All the differences discussed above clearly support the recognition of the specimens belonging to *Clavellotis* from sparid fish of the Yucatán Peninsula as a distinct species, herein described as *Clavellotis mayae* n. sp., representing the first record of the genus on the Atlantic coast of Mexico, parasitizing sparid fish (*L. rhomboides, A. rhomboidalis* and *A. probatocephalus*).

### Molecular characterization

**Figure 6.**
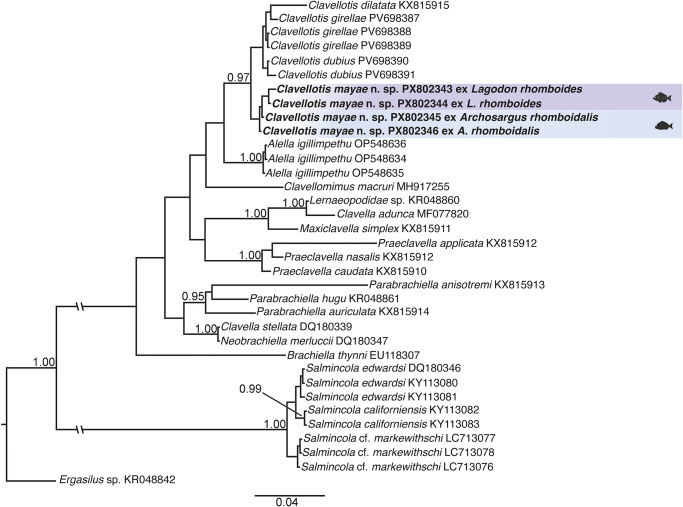
Phylogenetic relationships of *Clavellotis mayae* n. sp. (Copepoda: Lernaeopodidae) from *Lagodon rhomboides* (L.) and *Archosargus rhomboidalis* (L.) from the Yucatán Peninsula, Mexico, and other *Lernaeopodid copepod* species, obtained from Bayesian Inference (20 000 000 generations) of the 28S rDNA sequences. Branch support values correspond to posterior probabilities ≥0.90. Newly generated sequences are shown in bold. Figure 6 long description.

***28S rDNA.*** Partial 28S rDNA sequences were generated for 4 specimens of *C. mayae* n. sp. (686–1063bp) parasitizing *L. rhomboides* collected in La Carbonera lagoon and *A. rhomboidalis* in Celestún lagoon. The newly generated sequences were compared with homologous sequences of Lernaeopodidae available in GenBank (Table 1), the alignment was 482 bp long and contained 22 taxa. *Ergasilus* sp. (KR048842) was used as an outgroup. The sequences of *C. mayae* n. sp. nested with all species of *Clavellotis*, a relationship supported by high posterior probability values (0.97) (Figure 6). The intraspecific genetic divergence among isolates of *C. mayae* n. sp. was low, ranging from 0.42% to 1%, whereas the interspecific divergence varied between 1% and 3% with respect to the other congeners (Table 2). In the 28S phylogenetic tree the new species was recovered as the sister species of a clade containing *C. dubius* plus *C. girellae* and *C. dilatata,* the only members of the genus for which 28S rDNA sequences are available.

**Table 2.** Interspecific genetic distances among species included in the phylogenetic analyses, 28S rDNA distances below the diagonal; *cox*1 distances above the diagonal; distances are expressed in percentages. Newly generated sequences are shown in bold Table 2 long description.

	1	2	3	4	5	6	7	8	9	10	11	12	13	14	15	16	17	18	19	20	21	22
1 Outgroup		30	31		34			30	26	30	29	29	29	30	33			32	**29**	30	31	31
2 *Salmincola cf. markewithschi*	26		15		27			23	23	22	23	23	22	29	28			25	**26**	25	25	25
3 *S. californiensis*	26	1			28			24	23	23	25	24	24	29	27			25	**26**	26	26	26
4 *S. edwardsi*	26	1	1																			
5 *Brachiella thynni*	22	18	19	18				23	22	22	24	24	23	27	23			24	**21**	22	23	23
6. *Neobrachiella merluccii*	23	16	17	16	10																	
7 *Clavella stellata*	23	16	17	16	10	0																
8 *Parabrachiella auriculata*	23	17	18	17	10	4	4		17	14	21	20	20	25	27			22	**18**	19	20	20
9 *P. hugu*	22	16	17	17	12	6	6	5		16	19	17	17	23	24			22	**19**	17	20	20
10 *P. anisotremi*	27	22	22	21	15	11	11	10	11		17	18	18	24	25			20	**17**	17	19	19
11 *Praeclavella caudata*	24	17	17	17	11	8	8	9	8	12		9	11	22	24			19	**19**	17	18	18
12 *P. nasalis*	25	17	17	17	11	8	8	10	9	12	2		6	22	23			19	**18**	18	18	19
13 *P. applicata*	28	20	20	20	15	13	13	14	12	16	7	6		23	24			20	**17**	17	17	19
14 *Maxiclavella simplex*	24	17	16	16	11	8	8	9	9	13	8	9/	13		24			24	**24**	23	23	22
15 *Clavella adunca*	24	18	18	17	11	9	9	10	11	14	8	8	12	5				25	**24**	24	24	24
16 *Lernaeopodidae* gen. sp.	25	17	17	17	12	8	8	9	9	14	8	9	13	4	1							
17 *Clavellomimus macruri*	24	18	18	18	13	8	8	10	9	14	8	8	13	9	10	9						
18 *Alella igillimpethu*	24	16	17	16	12	7	7	9	8	13	5	6	10	7	9	8	7		**17**	19	19	20
19 *Clavellotis mayae* n. sp.	**23**	**16**	**16**	**16**	**12**	**5**	**5**	**7**	**6**	**13**	**6**	**6**	**10**	**7**	**10**	**8**	**7**	**5**		**12**	**13**	**14**
20 *C. dubius*	23	16	17	16	12	6	6	8	7	13	6	6	11	8	10	8	6	4	**1**		14	13
21 *C. girellae*	23	16	16	16	12	6	6	8	7	13	6	6	11	8	10	9	6	5	**1**	1		7
22 *C. dilatata*	24	15	16	15	12	6	6	8	7	11	6	7	11	8	10	10	7	4	**3**	2	2	

**Figure 7.**
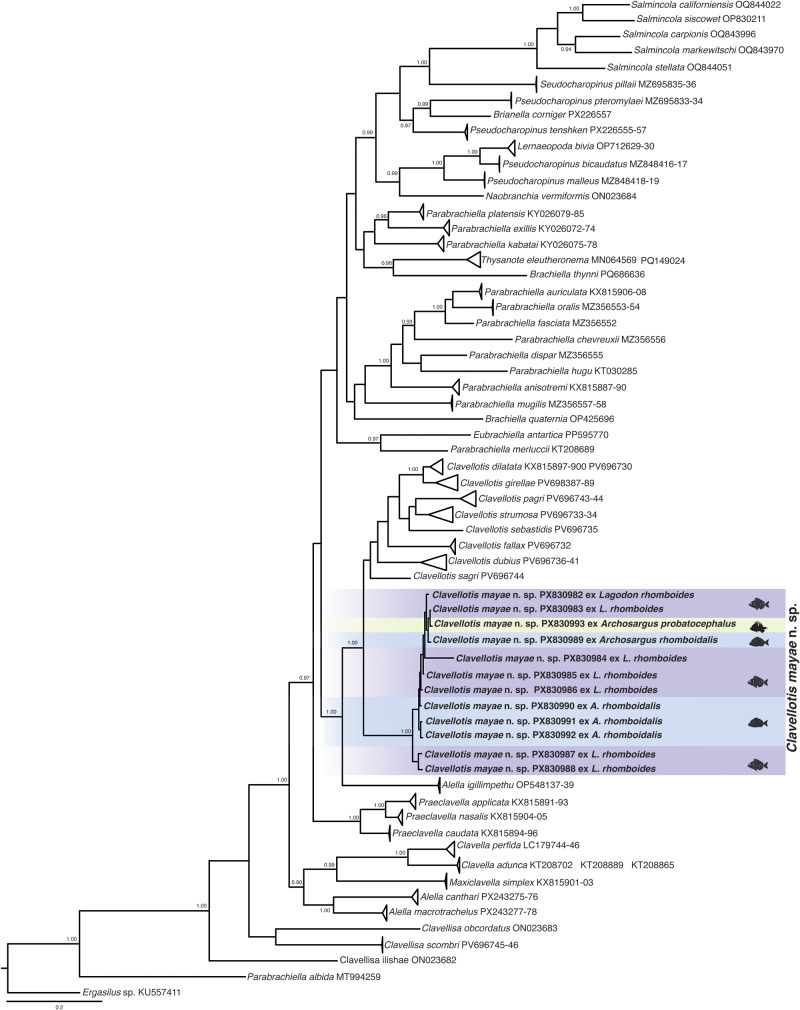
Phylogenetic relationships of *Clavellotis mayae* n. sp. (Copepoda: Lernaeopodidae) from *Lagodon rhomboides* (L.), *Archosargus rhomboidalis* (L.) and *Archosargus probatocephalus* (Walbaum) from the Yucatán Peninsula, Mexico, and other Lernaeopodid copepod species, obtained from Bayesian Inference (20 000 000 generations) of the *cox*1 mtDNA sequences. Branch support values correspond to posterior probabilities ≥0.90. Newly generated sequences are shown in bold. Figure 7 long description.

***cox*1 *mDNA.*** Partial *cox*1 mDNA sequences were generated for 12 specimens of *C. mayae* n. sp. collected from *L. rhomboides* (579–676 bp), *A. rhomboidalis* (472–651 bp) and *A. probatocephalus* (663 bp). The newly generated sequences were analysed together with sequences of Lernaeopodidae available in GenBank (Table 1), the alignment included 121 isolates. *Ergasilus* sp. (KR048842) was used as an outgroup. The 12 isolates of *C. mayae* n. sp. clustered together in a well-supported clade (PP = 1), as the sister taxa of a clade containing 8 species of *Clavellotis* for which *cox*1 is currently available (Figure 7). The intraspecific genetic divergence among isolates varied between 1% and 3% (Table 2). Instead, the *cox*1 interspecific divergence between *C. mayae* n. sp. and all the other species of *Clavellotis* varied from 12% to 14% (Table 2).

## Discussion

Morphological and molecular data corroborated that *Clavellotis mayae* n. sp. represents a new species. Morphologically, the new species shows closer affinities with species bearing an elongated or pyriform trunk, but differing from them by possessing a subcircular, orbicular, or quadrangular trunk and notably, by having a suboval aliform process together with lobules at the base of the maxilla; this represents a unique combination of characters not observed in any other *Clavellotis* species. In addition, the bases of the egg sacs are protected by trunk protrusions resembling flaps, a condition that further distinguishes the new species.

These morphological differences were corroborated by molecular data. DNA sequences support that all isolates for both molecular markers reveal the presence of a single species irrespective of the species of sparid they infect; molecular data also corroborated the validity of the new species considering the phylogenetic position on the trees, and the interspecific genetic divergence with respect to all the congeneric species for which sequences of the 2 molecular markers are available. In this case, the *cox*1 tree resulted to be more informative for species recognition since a larger number of species of *Clavellotis* have been sequenced; however, higher rank phylogenetic relationships contrast with the 28S tree, particularly regarding the monophyly of genera such as *Parabrachiella* which appears to be paraphyletic in the *cox*1 tree, whereas in the 28S tree it is recovered as monophyletic, although with a lower number of available sequences (Figures 6 and 7).

With the inclusion of the new species described herein from sparid hosts from the Yucatán Peninsula, Mexico, the total number of recognized species in the genus rises to 14. Species in the genus are widely distributed across the globe. Six species have been reported from the Mediterranean Sea, namely *C. briani, C. characis, C. fallax, C. sargi, C. strumosa* and *C. pagri*. Interestingly, *C. pagri* has been also reported from Argentinean and Brazilian waters, in the Southwestern Atlantic (Cantatore et al., 2012). Another 3 species were reported from the South Pacific, including *C. dilatata, C. dubius* and *C. girellae. Clavellotis sebastidis* apparently possesses a wider distribution range, since it has been reported from the South Atlantic and North Pacific waters (Castro and González, 2005; Rodríguez-Santiago et al., 2014, 2015, 2016). In addition, *C. bilobata* Pillai, 1962 was described from the Indian Ocean, *C. terakihi* from Australian waters, and *C. branchiostegui* from Japanese waters (Walter and Boxshall, 2025).

The validation of some species require further corroboration, such as the recently described *Clavellotis helicoleni* Izawa, 2025, since the male morphology does not conform with the diagnostic characters of *Clavellotis* and the female possesses small caudal rami which is absent in all other species. Also, some records from Japan require an integrative review, such as *C. nodula* Do & Ho, 1983, which has been proposed as a synonym of *C. dilatata*.

## Figure 1 Long description

The map illustrates the Yucatán Peninsula in Mexico, highlighting three specific sampling locations marked with stars near the Gulf of Mexico. The map includes latitude and longitude lines, with coordinates ranging from 20 degrees 0 minutes North to 22 degrees 0 minutes North and 87 degrees 0 minutes West to 91 degrees 0 minutes West. A scale bar indicates distances of 25 and 50 kilometers. An inset map in the top left corner shows the location of the Yucatán Peninsula within a broader map of Central America. A compass rose is present in the top right corner for orientation.

Navigate back to Figure 1.

## Figure 2 Long description

The image A shows the lateral view of the female Clavellotis mayae, featuring the cephalothorax labeled as Ce, maxilla labeled as m, aliform process labeled as ap, lateral lobes labeled as lb, trunk labeled as t and egg sac labeled as es. The image B shows the dorsal shield with the label de. The image C shows the maxilla and aliform process in lateral view, with labels m and ap. The image D shows the maxilla and aliform process in dorsal view, with labels lb and ap. Each illustration includes a scale bar for reference.

Navigate back to Figure 2.

## Figure 3 Long description

The image shows detailed anatomical illustrations labeled A to G. A depicts the trunk distal margin with features like flaps and genital process. B1 shows the entire antennule with a whip, while B2 details the distal armature with numbered setae. C1 illustrates the antenna with exopod and endopod and C2 provides a closer view of the endopod detail. D presents the labrum in ventral view with rostral seta. E shows the mandible with a serrated edge. F illustrates the maxillule with endopod and palp. G depicts the maxilliped with corpus, claw and accessory barb. Each illustration includes scale bars for reference.

Navigate back to Figure 3.

## Figure 4 Long description

The illustration shows various anatomical parts of the male n. p. from the Yucatán Peninsula, Mexico. A depicts the entire lateral view of the male, highlighting the genital process, maxilla and maxilliped. B1 shows the entire antennule, while B2 details the distal armature with setae labeled 1 to 6. C1 presents the entire antenna and C2 focuses on the distal endopod. D illustrates the mandible with a thin blade and eight equal teeth. E displays the maxillule with an endite and papillae bearing setae. F shows the maxilla with a globose first segment and curved claw. G provides dorsal and ventral views of the maxilliped, showing teeth on the segments. Scale bars are included for reference.

Navigate back to Figure 4.

## Figure 5 Long description

Image A shows a female n. sp. from the Yucatán Peninsula in an entire lateral view, displaying elongated body features. Image B presents the posterior end of the female with visible egg sacs, arranged in a linear pattern. Image C depicts a male attached to the female genital process, with labels indicating 'm' for male and 'gp' for genital process. Image D shows a male specimen, highlighting its distinct body shape. Each image includes a scale bar for reference.

Navigate back to Figure 5.

## Figure 6 Long description

The phylogenetic tree illustrates the relationships among different copepod species, focusing on Clavellotis mayae n. sp. The tree includes sequences from Clavellotis mayae n. sp. obtained from Lagodon rhomboides and Archosargus rhomboidalis. The tree shows branch support values, with posterior probabilities indicated, such as 0.97 for Clavellotis girellae and Clavellotis dubius. Ergasilus sp. KR048842 is used as an outgroup. The tree includes other species like Alella gilgilimpethu, Clavellomimus macruri and various Salmincola species. The scale bar at the bottom represents genetic distance, marked as 0.04.

Navigate back to Figure 6.

## Figure 7 Long description

The phylogenetic tree illustrates the relationships among various copepod species, with a focus on Clavellotis mayae n. sp. The tree includes sequences from different hosts: Lagodon rhomboides, Archosargus rhomboidalis and Archosargus probatocephalus. The highlighted section shows Clavellotis mayae n. sp. sequences, each labeled with specific identifiers and host names. The tree branches display support values, indicating the confidence in the relationships depicted. Various other species are listed with their respective identifiers, showing the broader phylogenetic context. The tree structure is detailed, with branches and nodes representing evolutionary connections among the species. Ergasilus sp. is used as an outgroup, providing a reference point for the tree's root.

Navigate back to Figure 7.

## Table 1 Long description

The table provides detailed information on various copepod species, including their host organisms, collection sites, and GenBank accession numbers for genetic sequences. Key data points include species like Alella canthari collected in Turkey and Clavellotis mayae in Mexico, with newly generated sequences emphasized in bold. The table also shows a variety of collection sites, such as Turkey, South Africa, and Chile, indicating a wide geographical distribution. Some entries lack complete data, such as missing host or site information, which may affect comprehensive analysis. The table serves as a resource for understanding the genetic diversity and distribution of copepods across different regions.

Navigate back to Table 1.

## Table 2 Long description

The table measures interspecific genetic distances among various species using 28S rDNA and cox1 gene sequences, expressed as percentages. Salmincola cf. markewithschi and S. californiensis exhibit the smallest cox1 genetic distance at 15%, indicating close genetic similarity. In contrast, Brachiella thynni and Neobrachiella merluccii show the largest 28S rDNA genetic distance at 18%, suggesting greater genetic divergence. Newly generated sequences are highlighted in bold, providing insights into recent genetic data. The table allows for comparison of genetic distances both within and between species, offering a comprehensive view of phylogenetic relationships. Interpretation should consider the variability in genetic markers and the potential impact of newly generated sequences on phylogenetic analysis.

Navigate back to Table 2.
